# Supplementary material for: Evaluation of altered patterns of tactile sensation in the diagnosis and monitoring of leprosy using the Semmes-Weinstein monofilaments
Source: PLoS One. 2022 Aug 10;17(8):e0272151. doi: 10.1371/journal.pone.0272151 (PMC9365170; doi:10.1371/journal.pone.0272151)
Supplement: S1 File — (PDF) [file pone.0272151.s001.pdf]

Results

Binomial Logistic Regression

Model Fit Measures

						Overall Model Test		
Model	Deviance	AIC	R^2_{McF}	R^2_{CS}	R^2_N	χ^2	df	p
1	54.2	64.2	0.418	0.401	0.568	39.0	4	< .001

Omnibus Likelihood Ratio Tests

Predictor	χ^2	df	p
Sex	0.0439	1	0.834
Age	0.4305	1	0.512
Points Pre	1.3764	1	0.241
Score	36.7002	1	< .001

[3]

Model Coefficients - Sensory recovery

		95% Confidence Interval						95% Confidence Interval	
Predictor	Estimate	Lower	Upper	SE	Z	p	Odds ratio	Lower	Upper
Intercept	-18.9481	-3571.8841	3533.9879	1812.7557	-0.0105	0.992	5.90e-9	0.000	Inf
Sex:									
1 – 0	0.1434	-1.2011	1.4879	0.6860	0.2090	0.834	1.154	0.301	4.43
Age	0.0139	-0.0281	0.0559	0.0214	0.6507	0.515	1.014	0.972	1.06
Points Pre	-0.0482	-0.1296	0.0333	0.0415	-1.1595	0.246	0.953	0.878	1.03
Score	19.3052	-3533.6303	3572.2406	1812.7555	0.0106	0.992	2.42e+8	0.000	Inf

Note. Estimates represent the log odds of "Sensory recovery = 1" vs. "Sensory recovery = 0"

Assumption Checks

Collinearity Statistics

	VIF	Tolerance
Sex	1.06	0.945
Age	1.30	0.767
Points Pre	1.37	0.730
Score	1.00	1.000

[3]

Prediction

Cut-Off Plot

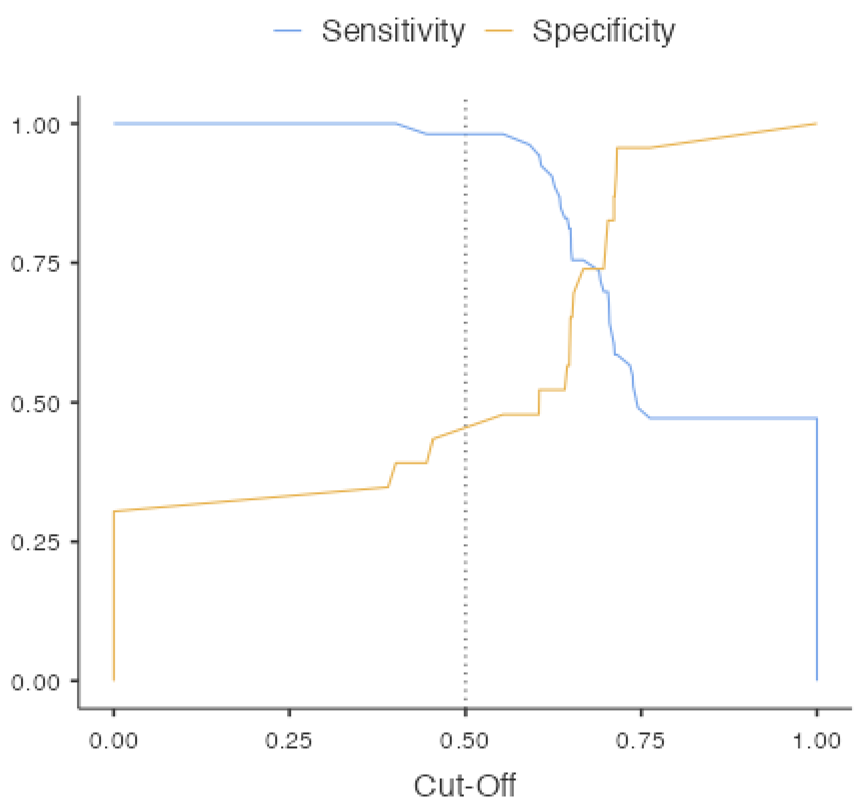

Predictive Measures

Accuracy	Specificity	Sensitivity	AUC
0.829	0.478	0.981	0.848

Note. The cut-off value is set to 0.5

ROC Curve

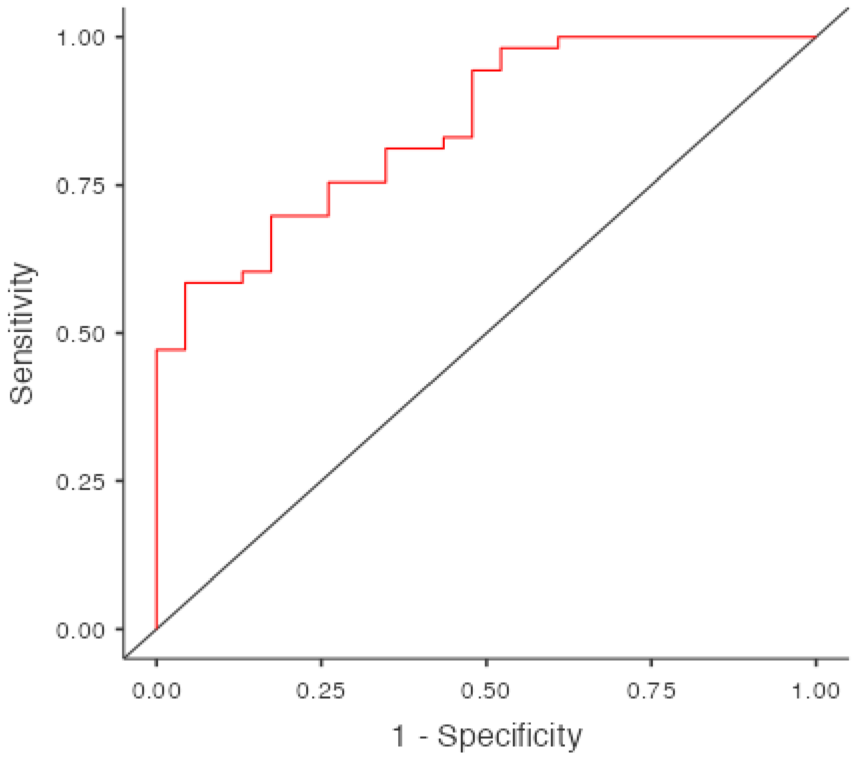

[4]

References

[1] The jamovi project (2021). *jamovi*. (Version 1.6) [Computer Software]. Retrieved from https://www.jamovi.org.

[2] R Core Team (2020). *R: A Language and environment for statistical computing*. (Version 4.0) [Computer software]. Retrieved from https://cran.r-project.org. (R packages retrieved from MRAN snapshot 2020-08-24).

[3] Fox, J., & Weisberg, S. (2020). *car: Companion to Applied Regression*. [R package]. Retrieved from https://cran.r-project.org/package=car.

[4] Sing, T., Sander, O., Beerenwinkel, N., & Lengauer, T. (2015). *ROCR: Visualizing the Performance of Scoring Classifiers*. [R package]. Retrieved from https://cran.r-project.org/package=ROCR.
