# Supplementary material for: Evaluation of altered patterns of tactile sensation in the diagnosis and monitoring of leprosy using the Semmes-Weinstein monofilaments
Source: PLoS One. 2022 Aug 10;17(8):e0272151. doi: 10.1371/journal.pone.0272151 (PMC9365170; doi:10.1371/journal.pone.0272151)
Supplement: Attachment — Submitted filename: Author Responses Aug 25, 2021.docx [file pone.0272151.s003.docx]

**Comments to the Author**

1. Is the manuscript technically sound, and do the data support the conclusions?

The manuscript must describe a technically sound piece of scientific research with data that supports the conclusions. Experiments must have been conducted rigorously, with appropriate controls, replication, and sample sizes. The conclusions must be drawn appropriately based on the data presented.

Reviewer #1: Yes

Reviewer #2: Yes

2. Has the statistical analysis been performed appropriately and rigorously?

Reviewer #1: Yes

Reviewer #2: I Don't Know

3. Have the authors made all data underlying the findings in their manuscript fully available?

The PLOS Data policy requires authors to make all data underlying the findings described in their manuscript fully available without restriction, with rare exception (please refer to the Data Availability Statement in the manuscript PDF file). The data should be provided as part of the manuscript or its supporting information, or deposited to a public repository. For example, in addition to summary statistics, the data points behind means, medians and variance measures should be available. If there are restrictions on publicly sharing data—e.g. participant privacy or use of data from a third party—those must be specified.

Reviewer #1: Yes

Reviewer #2: Yes

4. Is the manuscript presented in an intelligible fashion and written in standard English?

PLOS ONE does not copyedit accepted manuscripts, so the language in submitted articles must be clear, correct, and unambiguous. Any typographical or grammatical errors should be corrected at revision, so please note any specific errors here.

Reviewer #1: Yes

Reviewer #2: No

5. Review Comments to the Author

Please use the space provided to explain your answers to the questions above. You may also include additional comments for the author, including concerns about dual publication, research ethics, or publication ethics. (Please upload your review as an attachment if it exceeds 20,000 characters)

Reviewer #1: Congratulation to the authors for their study “Semmes-Weinstein Monofilament esthesiometry for the definition of patterns of sensitivity alteration of the hands and feet in leprosy diagnosis and monitoring”. Overall the manuscript is insightful and contributes to the subject of nervous impairment in leprosy patients, specifically MB. The study is very detailed and meticulous. It addresses some gaps found in literature. However, we do have some concerns worth addressing:

1. You state repeatedly throughout the manuscript that you only included multibacillary (or “lepromatous”) cases of leprosy in the study. However, Table 2 details how 101 patients from the initial population were actually classified as Borderline. Therefore, I must inquire:

- Which diagnostic criteria did you use to define the patients? The WHO classification, The Ridley-Jopling classification? (PMID: 17366457) Please define in Materials and Methods, for the sake of clarity.

Author’s comments: Sure, you are right. We did not consider describe about it in the manuscript. In lines 102 to 109, we have added:

Diagnostic criteria for leprosy

The subjects underwent a standardized clinical dermatoneurological exam according to Brazilian Ministry of Health guidelines as described in previous article from our group. [16,17]. We classified the patients considering the guidelines adapted by Madrid (Congress of Madrid 1953) [18] and the Indian Association of Leprology (IAL 1982) [19] classifications, as follows: indeterminate (I), polar tuberculoid (T), borderline (B), polar lepromatous (L) and pure neural leprosy (PNL); and broadly classified according to WHO operational criteria [PB (I and T) and MB (B and L)] [16,20].

- Why did you decide not to include patients with paucibacillary leprosy in this study? This should also be stated in the manuscript for the sake of transparency. Perhaps include it as one of the limits of the study.

Author’s comments: Thanks for your comment. Following the classification criteria based on both cutaneous and nerve evaluations by the specialist, the clinical classification of the patients as paucibacillary become difficult because most of the patients presented more than one nerve impairment added to skin signs of leprosy.

2. You stress the importance of tibial involvement in leprosy, which is not something new to date. You should consider better implementing what is already known in literature and discuss it in the manuscript (PMID: 4795534, which is also specific for MB patients, and PMID: 11391190).

Author’s comments: Thanks for your comment. We highlighted the importance of the tibial involvement in leprosy patients as compared to other nerves mainly to upper limbs. Both articles that you mentioned described interesting studies about tibial nerve exclusively different of our goal to show the importance of esthesiometric tool for the timely diagnosis of leprosy.

About the papers, the first paper by Swift et al (1973), they studied only about the motor conduction velocity, and they did not study the sensory nerves. On the other hand, on the paper from Richard et al (2001), they selected nine leprosy patients with exclusive tibial nerve palsy causing a loss of protective sensation on the sole of the foot, and they considered a very late loss of sensation defined only when they not feeling a 10-g monofilament in at least three sites on the foot.

3. You analyse the esthesiometric improvement in the tested nerves after treatment with WHO regimen. However, not all included patients started with the same severity. In fact, you should consider implementing a multivariate statistical analysis to understand the weight of severity on sensory recovery after treatment. Perhaps also consider evaluating the effects of age and gender as well.

Author’s comments: We thank you for your considerations to improve our paper. We have made the multivariate statistical analysis using a binomial logistic regression analysis, and to get a good performance of this statistical model we considered as independent variables age, sex, number of altered esthesiometric points in pre-treatment, the score of sensory recovery (0 to 5 according each five recovered points), and finally as outcome the esthesiometric clinical improvement (dependent variable) which it was established by the difference between the summation of the number of pre-treatment altered points and the post-treatment ones becoming to nominal variable.

The logistic regression model was significant (X^2^=39.0; p<0.001; R^2^ MacFadden=0.41; Accuracy=0.82; Specificity=0.47; Sensitivity=0.98; AUC=0.84), demonstrating an absence of association between age [OR=1.01 (%CI=0.97-1.06); p=0.5], sex [OR=1.1 (%CI=0.3-4.4); p=0.8], number of pre-treatment altered esthesiometric points [OR=0.9 (%CI=0.8-1.03); p=0.2] with the clinical outcome (esthesiometric clinical improvement). Therefore, independent of weight of severity of number of altered esthesiometric points in pre-treatment, after the treatment the sensory recovering happened.

In summary, we improved the manuscript adding:

1)	Statistical analysis topic: lines 228-231: “The Binomial Logistic Regression Analysis was performed in order to assess the association age, sex and number of pre-treatment altered esthesiometric points with the outcome of having a esthesiometric clinical improvement using the jamovi project (2021). *jamovi* (Version 1.6) [Computer Software]. Retrieved from https://www.jamovi.org.”

2)	Results topic: lines 410-414: “The logistic regression model was significant (X^2^=39.0; p<0.001; R^2^ MacFadden=0.41; Accuracy=0.82; Specificity=0.47; Sensitivity=0.98; AUC=0.84), demonstrating an absence of association between age [OR=1.01 (%CI=0.97-1.06); p=0.5], sex [OR=1.1 (%CI=0.3-4.4); p=0.8], number of pre-treatment altered esthesiometric points [OR=0.9 (%CI=0.8-1.03); p=0.2] with the clinical outcome (esthesiometric clinical improvement).”

3)	In the end of the discussion topic: lines 533-535. “…Furthermore, considering the binomial logistic regression analysis, we emphasize that regardless of the initial severity weight based on the number of altered esthesiometric points, the treatment was undoubtedly able to recover the sensitivity of hands and feet.”

4. You should better discuss the limitations in the study’s methodology. Specifically, you should at least mention alternatives that have been deemed superior in esthesiometric assessments, even if for different purposes, such as the PSSD (PMID: 26220428, PMID: 32128706).

Author’s comments: Sure, you are right. We mentioned about this method to assess the sensitivity as PSSD as described by Baltodano et al (2015) in the second paragraph of discussion. (lines 426-429).

We have added the (red) test below:

“…Although there is other device to assess the sensitivity for diagnosis and the follow-up of the neuropathy as the PSSD (pressure-specified sensory device), described by Baltodano et al (2015), with perhaps a marginal improvement over the SWM, the major advantages using Semmes-Weinstein monofilaments are: its easy application, low cost and the fact that the patient is blindfolded, making it difficult to have false responses in the test [27].”

5. The way serology and anti-PDL-1 positivity affected esthesiometric changes is unclear. Although it is true that some evidence suggests that it could be a useful tool for evaluating the efficacy of treatment (i.e. higher serology levels in untreated patients) [PMID: 19784481], one must wonder how relevant is this section for the purpose of your study? Especially considering that correlation index was found to be low.

Author’s comments: Thanks for your comments. About this point of view, we highlighted that we found around 82% of patients (Table 6) with some altered esthesiometric point while the anti-PGL-I positivity was 40% only (Table 5).

6. Some minor critiques to the content of the manuscript should also be addressed:

• Consider implementing the type of study (i.e. cohort study) in the title of the manuscript;

Author’s comments: Thank you so much about this comment. Really, this new title will improve the quality and importance of our work. We changed the title to:

“Semmes-Weinstein Monofilament esthesiometry for the definition of patterns of sensitivity alteration of the hands and feet in a cohort leprosy study”

• Introduction is not engaging enough, too long and too focused on academic concepts which add very little to the manuscript. Consider reworking it in order to make it more focused on the aims and endpoints of the study;

Author’s comments: Thanks for your comment. We agreed with you. We cut the firs paragraph completely and changed a bit the second one. Additionally, considering the comment below, we moved the cited paragraph to the discussion part.

• Consider moving the following sections of the manuscript to the Discussion: “In a series of studies […] fibular nerve and the median” (p. 4, lines 75-80);

Author’s comments: Answered above.

• Consider moving the following sections of the manuscript to the Materials and Methods: “The standard esthesiometer kit […] the dermatomes of each nerve” (p. 4-5, lines 84-92); “All patients were treated […] + clofazimine 50 mg” (p. 26, lines 474-477).

Author’s comments: Thanks a lot. We have moved them for the methods.

• Finally, some very minor language concerns: “focallity” instead of focality (p.3, line 44); “surreal” instead of sural (p. 22, line 375). Please do consider using a native English speaker for a quick grammar and spelling check.

Author’s comments: Thanks a lot. We changed them.

Reviewer #2:

1)there is very little that is new in in this manuscript. The SWM(S"deemmes-Weinstein monofilament)and ball point pen and the PSSD (pressure-specified sensory device) have been reported almost endlessly to show that medical management is effective. This study does confirm these classic findings , and yet there is no reference to this, espeially not even one mention of the PSSD, which has been shown to be more sensitive in identifying early cases of Leprosy. At the end, I will include a few references that must be added

Author’s comments: Sure, thanks a lot for your comment. We mentioned about this method to assess the sensitivity as PSSD as described by Baltodano et al (2015) in the second paragraph of discussion. (lines 426-429).

We have added the (red) test below:

“…Although there is other device to assess the sensitivity for diagnosis and the follow-up of the neuropathy as the PSSD (pressure-specified sensory device), described by Baltodano et al (2015), with perhaps a marginal improvement over the SWM, the major advantages using Semmes-Weinstein monofilaments are: its easy application, low cost and the fact that the patient is blindfolded, making it difficult to have false responses in the test [27].”

2) I am sorry to point out all that the SWM estimates the sensibility of a point of skin. it does not make a true measurement. As this paper proves, a set of 6 filaments were used, and therefore there are no normative data but an estimate of a range. Did each filament chosen as the correct one for that point of skin have the number of the filament,, for example 2.83, first converted from its logarithmic form before statistics That number on the filament is the force (not pressure) of that piece of skin and is the log to the base 10 of that force in 0.1mg. So if pressure is going to be discussed, each measurement of force must be changed to pressure by dividing by the diameter of the filament used.

Author’s comments: Thanks a lot. We agree with you, but some points should be clarified according to the references below:

The SWM test uses a subjective approach to evaluate the threshold of sensitivity of an area of skin: “does the patient perceive the application of a standard filament of specific critical force?” The critical force is the axial force necessary to cause the filament to buckle. The pressure over a nerve ending or group can be estimated, if necessary, for purposes of comparison, either by dividing the filament’s critical force by the area of the tip of the filament in contact with the skin during application, (possibly varying during application) or by the estimated area of skin which is effectively depressed over the relevant nerve endings during the application. Traditionally the calculation is simply Critical Force / Tip Area.

The use of the logarithmic Manufacturer´s filament Number (MN) was shown at least as early as 1978 (Levin S, et al.) to be a source of confusion (these authors also pointed out that an engineering approach would require Stress rather than Pressure as the relevant variable), and was only introduced (by Weinstein) to facilitate the graphic visualization and statistical treatment of results. It certainly does not help to convert the traditional MN numbers back to gram force values as they have long become irrelevant. For example, the widely used 10 gram-force filament, chosen by Birke and Sims in 1986 to identify the threshold between presence or absence of protective sensation in the plantar surface of the foot, would have to be renumbered “5.00” instead of the much-vaunted “5.07”, which is the log value for 11.749 grams-force. Incidentally, the filament cited in that paper as “75 grams” is quoted as having the MN 6.10, which would suggest that they may have been using a 125 gram filament.

On the other hand, Bell-Krotoski and others have long recommended the simple use of the standard monofilament force values as they have repeatedly been shown to provide consistent and useful results for clinical evaluations and follow-ups over time. The pressure applied or stress does not need to be imposed as if it were a more scientific measure.

LEVIN, S; PEARSALL, G; RUDERMAN, R J. Von Frey's method of measuring pressure sensibility in the hand: an engineering analysis of the Weinstein-Semmes pressure aesthesiometer. The Journal of hand surgery, v. 3, n. 3, p. 211-216, 1978.

BIRKE, J. A.; SIMS, D. S. Plantar sensory threshold in the ulcerative foot. Leprosy review, v. 57, n. 3, p. 261-267, 1986.

PFAU, D. B., HAROUN, O., LOCKWOOD, D. N., MAIER, C., SCHMITTER, M., VOLLERT, J., ... & TREEDE, R. D. Mechanical detection and pain thresholds: comparability of devices using stepped and ramped stimuli. Pain Reports, 5(6), 2020.

BELL-KROTOSKI, J., & TOMANCIK, E. The repeatability of testing with Semmes-Weinstein monofilaments. The Journal of hand surgery, 12(1), 155-161. 1987.

3) The SWM measures sensibility of a piece of skin and yet the authors refer to these as dermatomes. Clearly they are measuring a piece of the skin territory of a peripheral nerve, and not a dermatome. See Figures 1 and 2 for example as well as the text.

Author’s comments: Thanks for your comments. We changed the manuscript to become clear our message about the esthesiometry.

Lines 130-132: With the patient’s eyes closed, each monofilament was applied perpendicularly for Lines 151-157: about 1 to 2 seconds at each skin point inside the respective dermatome area.

We changed the title of the Figures 1 and 2.

4) rather paper should say skin pressure threshold for one point static touch was estimated with the SWM.

Author’s comments: Sure, we have added this message into the paragraph starting in line 127

“Considering the skin pressure threshold for one-point static touch was estimated with the SWM. Initially, the test with the monofilaments was demonstrated to the patient in an arm area with normal sensitivity. After this stage, the test began with the SW monofilaments. With the patient’s eyes closed, each monofilament was applied perpendicularly for about 1 to 2 seconds at each skin point inside the respective nerve sensitivity territory (dermatome).”

when the above is cleared up, and tables, corrected, and numerical pressure not force values are used, I am happy to reconsider this paper

Author’s comments: We understood your point of view, but about it, we clarified in Methods (lines 142-149) why we have used the standard monofilament force values as proposed by Bell-Krotoski et al (1987).

here are recent articles showing what these authors show with medical treatment, but these apply to nerve decompression, which is the disability these patients have after medical treatment (also not mentioned in this paper)

Baltodano, PA, Wan, E, Noboa, J, Rosson, GD, Dellon, AL, Selecting a Test for

Leprous Neuropathy Screening, J Reconstr Microsurg, 31:607-613, 2015.

Wan, E, Rivadeniera, AF, Serrano, HA, Napit, I, Garbino, JA, Joshua, J, Cardona-Castro, N,

Dellon, AL, Theuvenet, W, Protocol for a randomized controlled trial investigating

decompression for leprous neuropathy (the DELN protocol), Lepr Rev, 87:553-561,2016.

Wan, EL, Noboa, J., Baltodono, PA, Jousin, RM, Erickson, W, Wilton, JP, Rosson, GD,

Dellon, AL, Nerve decompression for leprous neuropathy: A prospective study from

Ecuador, Lepr Review, 88:95-108, 2017.

Author’s comments: Thanks a lot. We have cited Baltodano et al (2015) above and we included the last ones changing the last paragraph of discussion to:

“In addition, the reduction in the values of these averages after the end of multidrug therapy demonstrates to what extent the treatment was able to modify the sensitivity profile, especially of the feet of these patients, and the most marked improvement being was evident among patients with fewer affected limbs, reaffirming the proposition that early diagnosis and treatment are essential in preventing deformities and disability, avoiding the need for decompression surgery to prevent neural damage in leprosy as described by Wan, EL et al (2016 and 2017).”

Thank you for your consideration. I look forward to hearing from you.

Sincerely,

**Statistical report**
