# Supplementary material for: Evaluation of altered patterns of tactile sensation in the diagnosis and monitoring of leprosy using the Semmes-Weinstein monofilaments
Source: PLoS One. 2022 Aug 10;17(8):e0272151. doi: 10.1371/journal.pone.0272151 (PMC9365170; doi:10.1371/journal.pone.0272151)
Supplement: Attachment — Submitted filename: Author Responses JAN 22, 2022.docx [file pone.0272151.s004.docx]

January 22, 2022.

Fabio Santanelli, di Pompeo d'Illasi, MD, PhD

Academic Editor

PLOS ONE

Sub: PONE-D-21-17910

Dear Editors,

We would like to thank you for all comments about our manuscript with changed title already: “Semmes-Weinstein Monofilament esthesiometry for the definition of patterns of sensitivity alteration of the hands and feet in leprosy diagnosis and monitoring” because they appointed some important aspects to improve it.

The requested edits were considered, and they are in a red in the manuscript.

**Answers to the editor about the reviewers’ comments**

1. If the authors have adequately addressed your comments raised in a previous round of review and you feel that this manuscript is now acceptable for publication, you may indicate that here to bypass the “Comments to the Author” section, enter your conflict of interest statement in the “Confidential to Editor” section, and submit your "Accept" recommendation.

Reviewer #2: (No Response)

 2. Is the manuscript technically sound, and do the data support the conclusions?

The manuscript must describe a technically sound piece of scientific research with data that supports the conclusions. Experiments must have been conducted rigorously, with appropriate controls, replication, and sample sizes. The conclusions must be drawn appropriately based on the data presented.

 Reviewer #2: No

3. Has the statistical analysis been performed appropriately and rigorously?

 Reviewer #2: No

4. Have the authors made all data underlying the findings in their manuscript fully available?

The [PLOS Data policy](http://www.plosone.org/static/policies.action#sharing) requires authors to make all data underlying the findings described in their manuscript fully available without restriction, with rare exception (please refer to the Data Availability Statement in the manuscript PDF file). The data should be provided as part of the manuscript or its supporting information, or deposited to a public repository. For example, in addition to summary statistics, the data points behind means, medians and variance measures should be available. If there are restrictions on publicly sharing data—e.g. participant privacy or use of data from a third party—those must be specified.

 Reviewer #2: Yes

5. Is the manuscript presented in an intelligible fashion and written in standard English?

PLOS ONE does not copyedit accepted manuscripts, so the language in submitted articles must be clear, correct, and unambiguous. Any typographical or grammatical errors should be corrected at revision, so please note any specific errors here.

 Reviewer #2: No

6. Review Comments to the Author

Please use the space provided to explain your answers to the questions above. You may also include additional comments for the author, including concerns about dual publication, research ethics, or publication ethics. (Please upload your review as an attachment if it exceeds 20,000 characters)

Reviewer #2: SWM “esthesiometry” Leprosy Rev 2021

1. The title of the paper and throughout the paper misuses “esthesiometry” and “sensitivity” and these need to be changed throughout the paper.

The SWM estimate a range for one-point static touch sensibility, not sensitivity.
Esthesiometry is the measurement of tactile sensibility.

The SWM cannot measure tactile sensibility and hence they cannot do esthesiometry,
even if this how the literature has misused these terms in the past. Time for perhaps
this paper to use the terms correctly.

Their title therefore might be: Evaluation of altered patterns of touch sensibility in
Leprosy using the Semmes-Weinstein monofilaments, and short title might me Patterns
of Sensibility in Hands and Feet of Leprosy Patients.

Author’s comments: Thanks a lot, to advertise us about our writing and meanings. We have changed the title for: “**Evaluation of altered patterns of tactile sensation in the diagnosis and monitoring of leprosy using the Semmes-Weinstein monofilaments”** becoming shorter than before and maintained the main idea. Also, we changed the short title as your suggestion to: **Hands and feet sensation patterns in leprosy**. Therefore, as we have used in our works before in neuropathy field, we used the term “sensitivity” (the quality or condition of being sensitive), instead of “sensibility” (the ability to appreciate and respond to complex emotional or aesthetic influences). By the way, the term “sensation” is used very frequently also, and we decided to change for. On the other hand, we would like to maintain “diagnosis and monitoring” because we have used SWM for monitoring leprosy neuropathy during the leprosy treatment a long time, but our data demonstrated that the SWM also can help the health professionals to stablish the pattern of the leprosy neuropathy diagnosis.

We have changed where we were using “esthesiometry” to SWM-test in all manuscript. Also, we have changed the using of the adjective “Esthesiometric” to SMW-test preceding the nouns throughout the paper marked in **red writing**.

Initially, in **lines 99-100, w**e excluded the sentence “also known as an esthesiometer” becoming: “Semmes-Weinstein monofilaments (SWM) are used to assess and monitor tactile sensation in specific territories of the nerve trunks of the hands and feet.”

In other important point, in Methods (page 6 – lines 153-154) we changed the subtitle **Esthesiometry** for “**Tactile sensation test by Semmes-Weinstein Monofilaments (SWM-test)**” to emphasize the correct meaning according to your suggestions (thanks again).

2. Abstract: This sensation is an example of how difficult it is to express what the authors are trying to say. They state “a 18% improvement in tactile sensitivity for the hands, and 28.7% for the feet was detected.” What can this possibly mean. Do they mean that 18% of their total number of hand patients had an improvement in sensation, or do they mean that the pre-treatment mean measurement has improved 18% for the hand.

Author’s comments: Thanks for bring us your difficulty to understanding our test. We are sure they will become our work better. In the abstract we changed this part for:

“At diagnosis, 81/107 (75.7%) patients had some degree of functional disability, and 46 (43%) of them had altered SWM-test in the hands and 94 (87.9%) of them in the feet. After one year of multibacillary multidrug therapy, the disability decreasing to 44/76 patients (57.9%) and decreasing of the percentual of altered SWM-test to 18% for the hands, and to 28.7% for the feet.”

Also, in the summary we have changed these considerations (marked in yellow):

“At diagnosis, 43% of them had altered SWM-test in the hands, while 87.9% had it in the feet. After one year of multibacillary multidrug therapy, the percentual of patients with altered SWM-test decreased to 18% for the hands and 28.7% for the feet.

3. Abstract: the next line is very confusing also: “In the hands, by esthesiometry, radial nerve was significantly asymmetric, while in the feet, the difference between the sum of altered esthesiometric points showed significant asymmetry when comparing both sides, highlighting the tibial nerve for the establishment of asymmetric leprosy neuropathy.” Can even the authors understand what this means????

Author’s comments: Thanks for your comment. We changed the test to be clearer and more understandable.

“In the hands, by SWM-test, only the radial nerve point demonstrated a significant asymmetry, while in the feet, the difference between the sum of altered SWM-test points showed significant asymmetry between both sides, highlighting the tibial nerve for the establishment of asymmetric leprosy neuropathy.”

4. The word “sensitivity” is still in this manuscript. Sensitivity is a statistical term as in sensitivity versus specificity. They use it wrongly when they should be saying sensibility.

Author’s comments: We understand your question, but we must disagree about your statement, as the term SENSITIVITY is not exclusive to statistics. According to several works in neuropathy, including some of our group, we have published it as SENSITIVITY, although some reviewers suggest that we change to "sensation", much more frequent than SENSIBILITY.

1. Frade MAC, Rosa DJF, Bernardes-Filho F, Spencer JS, Foss NT. Semmes-Weinstein monofilament: A tool to quantify skin sensation in macular lesions for leprosy diagnosis. Indian J Dermatol Venereol Leprol 2021; XX:1-9. (Added in our reference)
2. Fernanda Guzzo Gomes, Wilson Marques, Norma Tiraboschi Foss, Luísiane de Ávila Santana, Marco Andrey Cipriani Frade. Tactile threshold detection in leprosy patients with an electronic algometer. Journal of Neuroscience Methods, Volume 179, Issue 2, 2009, Pages 319-322,ISSN 0165-0270, https://doi.org/10.1016/j.jneumeth.2009.01.030.
3. Pedro J Tomaselli, Diogo F dos Santos, André C J dos Santos, Douglas E Antunes, Vanessa D Marques, Norma T Foss, Carolina L Moreira, Patrícia T B Nogueira, Osvaldo J M Nascimento, Luciano Neder, Amilton A Barreira, Marco A Frade, Isabela M B Goulart, Wilson Marques, Jr, Primary neural leprosy: clinical, neurophysiological and pathological presentation and progression, Brain, 2021;, awab396, <https://doi.org/10.1093/brain/awab396> (published in October 2021)

5. Conclusion: Sadly ,the authors FAIL to discus that the remaining disability these patients have is due to chronic nerve compression, which can be helped by surgical decompression. They must emphasize that while the r value of .80 is great after triple drug therapy, r2 = 64 which means that 36% of meaning is unexplained or not improved. Those patients after triple drug therapy have residual disability that appropriate peripheral nerve surgery can help. References to this fact were added as refs 34 and 35 at the very last lines of the Discussion, however only to say that drug therapy can reduce the need for surgery, rather than adding the remaining patients would benefit from surgery, and this needs to be emphasized.

a) Wan, EL, Rivadeniera, AF, Jousin, RM, Dellon, AL, Treatment of Peripheral Neuropathy in Leprosy: The case for nerve decompression, Plast Reconstr Surg GO, 2016; 4:e637; doi10.1097/GO0000000000000641.
b) b) Wan, E, Rivadeniera, AF, Serrano, HA, Napit, I, Garbino, JA, Joshua, J, Cardona-Castro, N, Dellon, AL, Theuvenet, W, Protocol for a randomized controlled trial investigating decompression for leprous neuropathy (the DELN protocol), Lepr Rev, 87:553-561, 2016.

Author’s comments: Thanks for your comment. We would like to say that now we understand you and we changed the last paragraph of the DISCUSSION to consider your point. In our conclusion, we considered difficult to include it because we did not consider this surgery procedure in our assistance unfortunately.

Lines 634-42: ….“It is worth emphasizing that those patients who, even after multidrug therapy, still have residual disability detected by the SWM test, may benefit from appropriate peripheral nerve decompression surgery, preventing definitive neural damage from leprosy, as described by Wan et al [34,35].”

Specifically in conclusion, we changed the writing explaining better about the SWM-test as a tactile sensitivity test:

Line 648 – “The tactile sensation test using Semmes-Weisntein monofilaments proved to be an important instrument…”

Line 673 – “Finally, hand and foot tactile sensation test using Semmes-Weinstein monofilaments proved to be effective…”

Additionally, we received recently (after our submission to the journal) one communication from the Brazilian health Ministry together SORRI, the company that produces the monofilament kits in Brazil about the change of the green monofilament from 0.05g to 0.07g recently adopted the new kit manual. According to the document below signed by Mr. Antony R. J. Nicholl, owner of the company with large expertise in SWM (we have made a free translation for English language), and considering that all our calculations were made considering values greater than 0.05 and as there is no value between 0.05 and 0.07, it has not really changed in our results and analysis, so we decided to do this up to date in our manuscript changing from 0.5 g-force to 0.07 g-force.

**In the manuscript we changed it in lines: 123, 136, 151, 159, 162, 167, 172, 180, 200 (Table 1), 255, 271, 290 and 495.**

Thank you for your consideration.

Sincerely,

Marco Andrey Frade

**SORRI'S ANSWER ON THE QUESTION ON THE CHANGE OF THE MONOFILAMENT FROM 0.05G TO 0.07G**

It is important to clarify that the filament, its composition, and its dimensions remain unchanged.

The green filament remains the same Nylon 612 filament, standardized by Semmes and Weinstein with an extrusion diameter of 5 thousandths of an inch and 38mm in length, and identified by them with the Logarithmic Marking Number of “**2.83**”, indicating a critical strength. buckling around **0.068** gf. The same filament that Weinstein found to be a good indicator of the threshold of normal tactile sensitivity in the hands.

The change is only the recognition that the filament initiates buckling upon application of an axial force of approximately **0.07** gf.

In the past, especially during the time of development of the summarized and portable set of 5, 6 and 7 filaments, as can be seen in several publications such as Bell-Krotoski (1990, 1993), Lehman (1993), the green filament, with its standard diameter of 5 thousandths of an inch, was known as “the 50 mg filament” – it seems to have become a nickname, but it does not represent a good approximation to the critical buckling force of the filament.

Following the recommendations of the pioneers in skin sensitivity mapping at the Leprosy Center in Carville, USA, the filaments used by SORRI-BAURU, in the manufacture of Stesiometer Kits, use the same type of nylon 612 and the same extrusion diameters used by Weinstein. and Semmes in their seminal studies in the 50s and 60s.

The filament selected to identify the threshold of sensitivity considered by them as “normal” for the trunk and upper limbs, especially the hands – that is, our green filament, was identified by Weinstein with the number “M = 2.83” – calculated as the common logarithm of 10 times the force in milligrams force required to initiate buckling of the filament mounted on a rigid rod, 38 mm free length.

M = log (10.Fmg)

Reversing the calculation, it can be seen that the critical force to initiate the filament buckling was, on average: 0.068 grams-force (or 68 milligrams-force).

Thus, for a long time, the nominal identification” of **0.05** g was perpetuated, recognizing, however, the value of **0.07 gf** as a better approximation of the value of the critical buckling force.

of the filament.

Regards,

Anthony RJ Nicholl,

1. Weinstein SW, Drozdenko R. Weinstein C: Some considerations of clinical tests. Presentation and discussion, Neuro Communication Research Laboratories, Inc., Danbury, CT, Sept 11,1992.

2. Weinstein S: Tactile sensitivity of the phalanges. Percept Mot Skills 14:351-354, 1962.

3. Weinstein S, Seren E: Tactile sensitivity as a function of handedness and laterality. J Comp Physiol Psychol 54:665-669, 1961.

4. Bell-Krotoski, J. (1990). “Pocket filaments” and specifications for the semmes-weinstein monofilaments. Journal of Hand Therapy, 3(1), 26-31.

5. Bell-Krotoski, J. A., Fess, E. E., Figarola, J. H., & Hiltz, D. (1995). Threshold detection and Semmes-Weinstein monofilaments. Journal of Hand Therapy, 8(2), 155-162.

6. Bowen, V. L.; Griener, J. S.; Jones, S. V. Threshold of sensation: Interrater reliability and establishment of normal using the Semmes-Weinstein monofilament. J Hand Ther, v. 3, p. 36-37, 1990.

7. Birke, J. A., Brandsma, J. W., Schreuders, T. A., & Piefer, A. (2000). Sensory testing with monofilaments in Hansen's disease and normal control subjects. International journal of leprosy and other mycobacterial diseases, 68(3), 291-298.

8. Naafs B, Dagne T: Sensory testing: A sensitive method in the follow-up of nerve involvement. Int J Lepr 45(4):364-368, 1977.

9. Lehman L F, Orsini M B P, Nicholl, A R J. The development and adaptation of the Semmes-Weinstein monofilaments in Brazil. Journal of Hand Therapy, v. 6, n. 4, p. 290-297, 1993.

10. Levin, S., Pearsall, G., & Ruderman, R. J. (1978). Von Frey's method of measuring pressure sensibility in the hand: an engineering analysis of the Weinstein-Semmes pressure aesthesiometer. The Journal of hand surgery, 3(3), 211-216.
